# Risk Factors, Management, and Outcome of Gastric Venous Congestion After Total Pancreatectomy: An Underestimated Complication Requiring Standardized Identification, Grading, and Management

**DOI:** 10.1245/s10434-023-13847-z

**Published:** 2023-08-19

**Authors:** Thomas F. Stoop, André von Gohren, Jennie Engstrand, Ernesto Sparrelid, Stefan Gilg, Marco Del Chiaro, Poya Ghorbani

**Affiliations:** 1 Division of Surgery, Department of Clinical Science, Intervention and Technology, Karolinska Institutet at Karolinska University Hospital, Stockholm, Sweden; 2 Amsterdam UMC, location University of Amsterdam, Department of Surgery, Amsterdam, The Netherlands; 3 Cancer Center Amsterdam, Amsterdam, The Netherlands; 4 Division of Surgical Oncology, Department of Surgery, University of Colorado Anschutz Medical Campus, Aurora, CO USA

## Abstract

**Background:**

Gastric venous congestion (GVC) after total pancreatectomy (TP) is rarely studied despite its high 5% to 28% incidence and possible association with mortality. This study aimed to provide insight about incidence, risk factors, management, and outcome of GVC after TP.

**Methods:**

This retrospective observational single-center study included all patients undergoing elective TP from 2008 to 2021. The exclusion criteria ruled out a history of gastric resection, concomitant (sub)total gastrectomy for oncologic indication(s) or celiac axis resection, and postoperative (sub)total gastrectomy for indication(s) other than GVC.

**Results:**

The study enrolled 268 patients. The in-hospital major morbidity (Clavien-Dindo grade ≥IIIa) rate was 28%, and the 90-day mortality rate was 3%. GVC was identified in 21% of patients, particularly occurring during index surgery (93%). Intraoperative GVC was managed with (sub)total gastrectomy for 55% of the patients. The major morbidity rate was higher for the patients with GVC (44% vs 24%; *p* = 0.003), whereas the 90-day mortality did not differ significantly (5% vs 3%; *p* = 0.406). The predictors for major morbidity were intraoperative GVC (odds ratio [OR], 2.207; 95% confidence interval [CI], 1.142–4.268) and high TP volume (> 20 TPs/year: OR, 0.360; 95% CI, 0.175–0.738). The predictors for GVC were portomesenteric venous resection (PVR) (OR, 2.103; 95% CI, 1.034–4.278) and left coronary vein ligation (OR, 11.858; 95% CI, 5.772–24.362).

**Conclusions:**

After TP, GVC is rather common (in 1 of 5 patients). GVC during index surgery is predictive for major morbidity, although not translating into higher mortality. Left coronary vein ligation and PVR are predictive for GVC, requiring vigilance during and after surgery, although gastric resection is not always necessary. More evidence on prevention, identification, classification, and management of GVC is needed.

During the last decade, a renewed interest in total pancreatectomy (TP) has evolved for various oncologic, technical, and safety indications.^1–3^ This interest is the consequence of improved surgical outcome for TP, particularly in high-volume centers.^4–7^ Furthermore, the endocrine and exocrine insufficiencies are more manageable currently,^8–11^ with the quality of life similar to that after partial pancreatectomy.^12,13^

A complication after TP that has been described but not fully explored is gastric venous congestion (GVC). Technically, a TP involves ligation of the right gastroepiploic vein, right coronary vein, splenic vein, short gastric veins, and left gastroepiploic vein when combined with concomitant splenectomy (or spleen preservation using the Warshaw technique). Occasionally, the left coronary vein is also ligated for oncologic and technical reasons. The subsequent impaired gastric venous outflow might lead to GVC, which in turn could result in gastric bleeding due to elevated intravascular pressure and stomach perforation because of hypoxia and necrosis by arterial stasis.

The literature about TP has expanded in recent years,^14,15^ but although GVC is described in some case reports,^16–19^ it is scarcely studied in consecutive series despite its seemingly high incidence of 5% to 28%^20–22^ and possible association with increased mortality.^21^ Therefore, the current study aimed to provide more insight into the incidence, risk factors, management, and outcome of GVC after TP.

## Methods

This retrospective single-center study was approved by the Ethical Committee Stockholm (Registration No. 2017/1977-32/1) and performed in accordance with the Strengthening the Reporting of Observational Studies in Epidemiology (STROBE) guideline.^23^

### Study Population and Design

The study enrolled all adult patients 18 years old or older who underwent an elective single-stage TP or an elective completion TP for any indication at Karolinska University Hospital from January 2008 to December 2021. The exclusion criteria ruled out TP with concomitant (sub)total gastrectomy for only an oncologic indication or during the postoperative course for gastro-enterostomosis leakage without evident venous drainage problems, a history of gastric resection, and concomitant celiac axis resection without preservation/reconstruction of the left gastric artery.

### Intraoperative Surgical Management

During the study period, there was no standardized strategy to prevent, identify (other than the surgeon’s visual judgement), or manage GVC at our institution. Intraoperatively, no gastroscopy, indocyanine green, flowmetry, or standardized surgical techniques (e.g., reconstruction of the left coronary vein or splenorenal shunts) were used.

### Definitions

Conditional status was classified using the American Society of Anesthesiologists–Physical Status (ASA-PS) classification.^24^ Total pancreatectomy and concomitant resections were defined in accordance with the International Study Group of Pancreatic Surgery (ISGPS) definition.^25^ Different types of portomesenteric venous resections (PVR) were classified according to the following ISGPS definitions: tangential resection with primary closure (type 1) or patch reconstruction (type 2), and segmental resection with end-to-end reconstruction (type 3) or using an interposition graft (type 4).^26^

In-hospital complications were collected. Pancreatic surgery-specific complications (i.e., delayed gastric emptying, postpancreatectomy hemorrhage [PPH], and bile leakage) were defined according to the ISGPS and International Study Group of Liver Surgery definitions, whereby only the clinically relevant grade B/C complications were registered.^27–29^ Major morbidity was defined as Clavien-Dindo grade IIIa or higher.^30^

Gastric venous congestion was defined as (1) congested gastric/perigastric veins with livid (bluish) stomach discolouring, (2) gastric wall edema, (3) petechial of the gastric serosa, and/or (4) congestion and/or hemorrhagic stomach mucosa as well as considerable venous bleeding when the stomach was opened.^21^ Because of the retrospective methodology, a description in the operation report indicating only GVC without further details also was considered sufficient for diagnosis. The diagnosis of GVC was determined intra- or postoperatively by computed tomography, endoscopy, and/or relaparotomy.^21^

Mortality was measured during hospitalization, then 90 days after index surgery. The annual elective single-stage and completion TP volumes were categorized together into low (< 10 TPs/year), moderate (10–20 TPs/year), or high (> 20 TPs/year). The elective TPs who did not meet the eligibility criteria from the current study were included for the yearly volume calculation.^5^ Histopathologic diagnoses were defined in accordance with the World Health Organization definition.^31^

### Statistical Analyses

Data analyses were performed with IBM ISPSS for Windows, version 26 (IBM Corp., Orchard Road Armonk, NY, USA). Statistical significance was considered at a two-tailed *p* value lower than 0.050. Categorical variables, analyzed with Pearson’s chi-square or Fisher’s exact test when appropriate, are presented as percentages and frequencies. The distribution of continuous data was assessed with histograms. Normally distributed continuous data, analyzed with the independent Student’s *t* test, are presented as means ± standard deviations (± SD). In contrast, non-normally distributed continuous variables, derived by analysis performed using the Mann-Whitney *U* test, are presented as medians with interquartile ranges (IQRs).

Logistic regression analysis was performed to investigate the potential predictors for major morbidity. The results are presented in odds ratios (ORs) with 95% confidence intervals (CIs).

Variables with a *p* value lower than 0.200 in the univariable analysis were further tested in the multivariable analysis.^32^ Subsequently, backward selection was performed until the multivariable model comprised only significant parameters (*p* < 0.050).

## Results

Overall, 287 patients underwent an elective TP during the study period. After exclusion of 19 patients (6.6%) because of (sub)total gastrectomy for only oncologic reasons (*n* = 16/19, 84.2%), history of gastric resection (*n* = 1/19, 5.3%), concomitant celiac axis resection without preservation/reconstruction of the left gastric artery (*n* = 2/19, 10.5%), and gastro-enterostomosis leakage without evidence of venous drainage problems (*n* = 0/19, 0%), 268 patients (93.4%) were included in the current analysis.

A total of 40 patients (14.9%) in the study cohort received preoperative chemotherapy ± radiotherapy. The majority of the patients underwent surgery for pancreatic adenocarcinoma (*n* = 140, 52.2%) or non-malignant intraductal papillary mucinous neoplasm (*n* = 47, 17.5%). (Table 1 presents the clinicopathologic characteristics).

**Table 1:** Baseline characteristics

Variable	All patients (*n* = 268)	Without GVC (*n* = 211)	With GVC (*n* = 57)	*p* value^a^
Age (years) (median, IQR)	69 (59–74)	69 (58–74)	70 (63–73)	0.627^b^
Female sex, *n* (%)	125 (46.6)	98 (46.4)	27 (47.4)	0.901^c^
BMI (kg/m^2^) (mean ± SD)	27 ± 14	27 ± 15	26 ± 4	0.724^d^
Comorbidities, *n* (%)	206 (76.9)	164 (77.7)	42 (73.7)	0.521^c^
Cardiovascular	133 (49.6)	102 (48.3)	31 (54.4)	0.418^c^
Hypertension	111 (41.4)	84 (39.8)	27 (47.4)	0.304^c^
Ischemic heart disease^e^	21 (7.8)	13 (6.2)	8 (14.0)	0.090^f^
Decompensatio cordis	7 (2.6)	6 (2.8)	1 (1.8)	1.000^f^
Peripheral vascular disease^g^	19 (7.1)	16 (7.6)	3 (5.3)	0.772^f^
Diabetes mellitus	74 (27.6)	58 (27.5)	16 (28.1)	0.931^c^
Chronic renal insufficiency	6 (2.2)	5 (2.4)	1 (1.8)	1.000^f^
Current smoker, *n* (%)	45 (16.8)	37 (17.5)	8 (14.0)	0.530^c^
History of malignancy, *n* (%)	62 (23.1)	48 (22.7)	14 (24.6)	0.773^c^
Preoperative chemo(radio)therapy, *n* (%)	40 (14.9)	26 (12.3)	14 (24.6)	**0.021**^c^
ASA–PS, *n* (%)				0.521^c^
I–II	165 (61.6)	132 (62.6)	33 (57.9)	
III–IV	103 (38.4)	79 (37.4)	24 (42.1)	
Postoperative diagnosis, *n* (%)				**< 0.001**^c,h^
Malignant	196 (73.1)	144 (68.2)	52 (91.2)	
Pancreatic adenocarcinoma	140 (52.2)	100 (47.4)	40 (70.2)	
Non-pancreatic periampullary adenocarcinoma	23 (8.6)	19 (9.0)	4 (7.0)	
Neuroendocrine tumor	15 (5.6)	11 (5.2)	4 (7.0)	
Metastases	14 (5.2)	11 (5.2)	3 (5.3)	
Others	3 (1.1)	2 (0.9)	1 (1.8)	
Non-malignant	72 (26.9)	67 (31.8)	5 (8.8)	
IPMN	47 (17.5)	46 (21.8)	1 (1.8)	
Chronic pancreatitis	21 (7.8)	18 (8.5)	3 (5.3)	
Others	5 (1.9)	4 (1.8)	1 (1.8)	

Bold value indicates statistical significance (*p* < 0.050)

GVC, gastric venous congestion; IQR, interquartile range; SD, standard deviation; BMI, body mass index; ASA-PS, American Society of Anesthesiologists–Physical Status; IPMN, intraductal papillary mucinous neoplasm

^a^Comparison between patients with and without GVC, ^b^Mann-Whitney *U* test, ^c^Chi-square test, ^d^Student’s *t* test, ^e^Including a history of angina pectoris, myocardial infarction, percutaneous coronary intervention and/or coronary artery bypass graft, ^f^Fisher’s exact test, ^g^Including vascular stenosis/aneurysm, transient ischemic attack and/or cerebrovascular accident, ^h^Malignant vs non-malignant

### Indications and Surgical Details

Most TPs concerned single-stage elective procedures (*n* = 232, 86.6%), the majority of which were initially scheduled as partial pancreatectomy but intraoperatively converted to TP (*n* = 148, 63.8%). No difference in conversion rate from partial pancreatectomy to TP was seen between the patients with GVC (*n* = 40/56, 71.4%) and those without GVC (*n* = 108/176, 61.4%) GVC (*p* = 0.172) (Appendix 1 presents the indications for intraoperative conversion).

Preservation of the spleen was performed for 13.1% of the patients. The remaining patients (*n* = 233, 86.9%) underwent a splenectomy with splenic vein ligation. The left coronary vein was ligated in 21.6% of the patients. Left coronary vein ligation was performed exclusively with splenic vein ligation (*n* = 58, 21.6%). The patients with GVC had higher rates of splenic vein ligation (*n* = 56/57 [98.2%] vs 177/211 [83.9%]; *p* = 0.004) and left coronary vein ligation (*n* = 36/57 [63.2%] vs *n* = 22/211 [10.4%]; *p* < 0.001) than those without GVC.

Almost half of the patients (42.9%) underwent an extended resection, particularly PVR (*n* = 93, 34.7%), followed by multivisceral resection (*n* = 46, 17.2%) and arterial resection (*n* = 25, 9.3%). The PVR procedure was performed more frequently in the GVC group (types 1 and 2 [*n* = 4/57, 7.0%]; types 3 and 4 [*n* = 30/57, 52.6%) than in the GVC group (types 1 and 2 [*n* = 8/211, 3.8%]; types 3 and 4 [*n* = 51/211, 24.2%; *p* < 0.001) (Table 2 presents the procedural details and differences between the patients with and without GVC).

**Table 2:** Procedural details

Variable	All patients (*n* = 268)	Without GVC (*n* = 211)	With GVC (*n* = 57)	*p* value^a^
TP type, *n* (%)				**0.004**^b^
Single-stage elective	232 (86.6)	176 (83.4)	56 (98.2)	
Elective completion	36 (13.4)	35 (16.6)	1 (1.8)	
*Standard procedural details*				
Auto-islet transplantation, *n* (%)	12 (4.5)	10 (4.7)	2 (3.5)	1.000^c^
Pylorus preservation, *n* (%)	90 (33.6)	85 (40.3)	5 (8.8)	**< 0.001**^b^
Spleen preservation, *n* (%)	35 (13.1)	34 (16.1)	1 (1.8)	**0.004**^b^
Ligation splenic vein, *n* (%)	233 (86.9)	177 (83.9)	56 (98.2)	**0.004**^b^
Ligation left coronary vein, *n* (%)	58 (21.6)	22 (10.4)	36 (63.2)	**< 0.001**^b^
Ligation splenic vein + left coronary vein, *n* (%)	58 (21.6)	22 (10.4)	36 (63.2)	**< 0.001**^b^
*Extended resections*				
Extended resection, *n* (%)	115 (42.9)	73 (34.6)	42 (73.7)	**< 0.001**^b^
Vascular resection, *n* (%)	100 (37.3)	65 (30.8)	35 (61.4)	**< 0.001**^b^
Portomesenteric venous resection				**< 0.001**^b^
Types 1–2	12 (4.5)	8 (3.8)	4 (7.0)	
Types 3–4	81 (30.2)	51 (24.2)	30 (52.6)	
Arterial resection^d^	25 (9.3)	19 (9.0)	6 (10.5)	0.726^b^
Hepatic artery	21 (7.8)	16 (7.6)	5 (8.8)	0.782^c^
Superior mesenteric artery	4 (1.5)	3 (1.4)	1 (1.8)	1.000^c^
Venous + arterial resection	17 (6.3)	12 (5.7)	5 (8.8)	0.371^c^
Multivisceral resection, *n* (%)	46 (17.2)	15 (7.1)	31 (54.4)	**< 0.001**^b^
(Sub)total gastrectomy	29 (10.8)	–	29 (50.9)	–
Colon resection	11 (4.1)	10 (4.7)	1 (1.8)	0.467^c^
Adrenalectomy	6 (2.2)	4 (1.9)	2 (3.5)	0.611^c^
Nephrectomy	5 (1.9)	3 (1.4)	2 (3.5)	0.288^c^
Liver resection	1 (0.4)	1 (0.5)	0 (0)	1.000^c^

Bold value indicates statistical significance (*p* < 0.050)

GVC, gastric venous congestion; TP, total pancreatectomy

^a^Comparison between patients with and without GVC, ^b^Chi-square test, ^c^Fisher’s exact test, ^d^All arterial resections are reconstructed

### Surgical Outcomes

The study identified GVC in 57 patients (21.3%). GVC was identified during index surgery in 93.0% (*n* = 53/57) of the patients, and during the postoperative course in the remaining 7.0% (*n* = 4/57) of the patients at a median of 16 days (IQR 8-38) postoperatively. In 54.7% (*n* = 29/53) of patients, the GVC was managed intraoperatively with a (sub)total gastrectomy. The remaining patients were treated conservatively.

No surgery was needed for the four patients whose GVC developed postoperatively. A diagnosis of GVC postoperatively was based on computed tomography (*n* = 1/4, 25.0%) or gastroscopy (*n* = 3/4, 75.0%). Three (75.0%) of the four patients with postoperative GVC presented with postoperative delayed gastric emptying grade B/C. In the remaining patient, GVC was diagnosed on computed tomography because of elevated laboratory infection parameters. In the four patients with postoperatively diagnosed GVC, no signs of GVC were present during the index surgery.

The overall major morbidity rate was 28.0% (*n* = 75). In a comparison of the patients with and without GVC, the major morbidity rate was higher for the patients who experienced GVC (*n* = 25/57 [43.9%] vs* n* = 50/211 [23.7%]; *p* = 0.003). Nevertheless, no statistical difference was seen in 90-day mortality (*n* = 3/57 [5.3%] vs* n* = 6/211 [2.8%]; *p* = 0.406). The 90-day mortality did not differ significantly between the patients with intraoperative GVC (*n* = 3/53, 5.7%) and those with postoperative GVC (*n* = 0/4, 0%) (*p* = 1.000) (Table 3 presents the perioperative outcomes and comparison of the patients with and without GVC).

**Table 3:** Perioperative outcomes

Variable	All patients (*n* = 268)	Without GVC (*n* = 211)	With GVC (*n* = 57)	*p* value^a^
*Intraoperative*				
Operation time (min) (mean ± SD)^b^	422 ± 112	401 ± 107	492 ± 101	**< 0.001**^c^
Intraoperative blood loss (ml) (median, IQR)^e,f^	350 (200–650)	350 (200–700)	380 (200–500)	0.762^d^
GVC, *n* (%)	53 (19.8)	–	53 (93.0)	–
GVC management				
Subtotal gastrectomy	27 (10.1)	–	27 (47.4)	–
Total gastrectomy	2 (0.7)	–	2 (3.5)	–
*Postoperative*				
GVC, *n* (%)	4 (1.5)	–	4 (7.0)	–
Any type of gastric resection	0 (0)	–	0 (0)	–
DGE grade B/C, *n* (%)^g^	37 (13.9)	28 (13.3)	9 (16.4)	0.555^h^
PPH grade B/C, *n* (%)	20 (7.5)	17 (8.1)	3 (5.3)	0.582^i^
Bile leakage grade B/C, *n* (%)	7 (2.6)	7 (3.3)	0 (0)	0.352^i^
Major morbidity, *n* (%)	75 (28.0)	50 (23.7)	25 (43.9)	**0.003**^h^
Intra-abdominal collection/abscess	27 (10.1)	19 (9.0)	8 (14.0)	0.263^h^
Gastroenterostomy leakage	3 (1.1)	3 (1.4)	0 (0)	1.000^i^
Wound dehiscence	7 (2.6)	3 (1.4)	4 (7.0)	**0.039**^i^
Relaparotomy	18 (6.7)	13 (6.2)^j^	5 (8.8)^k^	0.550^i^
Organ failure				0.080^i^
Single-organ failure	17 (6.3)	10 (4.7)	7 (12.3)	
Multi-organ failure	6 (2.2)	6 (2.8)	0 (0)	
Multiple major morbidity (> 1), *n* (%)	24 (9.0)	16 (7.6)	8 (14.0)	0.130^h^
Postoperative hospital stay (days) (median, IQR)	10 (8–14)	10 (8–14)	12 (8–16)	0.094^d^
Intensive care stay, *n* (%)	14 (5.2)	9 (4.3)	5 (8.8)	0.185^i^
Intensive care stay (days) (median, IQR)	4 (2–10)	7 (2–19)	2 (2–7)	0.219^d^
In-hospital mortality, *n* (%)	5 (1.9)	5 (2.4)	0 (0)	0.588^i^
90-day mortality, *n* (%)	9 (3.4)	6 (2.8)	3 (5.3)	0.406^i^

Bold value indicates statistical significance (*p* < 0.050)

GVC, gastric venous congestion; IQR, interquartile range; SD, standard deviation; min, minutes; DGE, delayed gastric emptying; PPH, postpancreatectomy hemorrhage

^a^Comparison between patients with and without GVC, ^b^Missing (*n* = 51), ^c^Student’s *t* test, ^d^Mann-Whitney *U* test, ^e^Missing data *n* = 21, ^f^Blood loss only from 2011 to 2021, ^g^Patients who underwent a total gastrectomy (*n* = 2) are excluded, ^h^Chi-square test, ^i^Fisher’s exact test, ^j^Indications for relaparotomy were gastroenterostomy leakage (*n* = 3), PPH (*n* = 3), wound dehiscence (*n* = 3), bile leakage (*n* = 6), ^k^Indications for relaparotomy were wound dehiscence (*n* = 4) and PPH (*n* = 1)

Logistic regression analysis identified a high volume (> 20 TPs/year: OR, 0.360; 95% CI, 0.175–0.738) as a protective predictor for major morbidity, whereas intraoperative GVC was found to be independently predictive for major morbidity (OR, 2.207; 95% CI, 1.142–4.268) (Table 4 presents the logistic regression analysis).

**Table 4:** Predictors for major morbidity

	Univariable analysis			Multivariable analysis		
Variable	OR	95% CI	*p* value	OR	95% CI	*p* value
Age (years)	1.018	0.993–1.042	0.155	–	–	–
BMI (kg/m^2^)	0.980	0.929–1.034	0.466	–	–	–
ASA-PS						
I–II (*n* = 165)		1 [Referent]		–	–	–
III–IV (*n* = 103)	1.736	1.010–2.983	**0.046**	–	–	–
TP type						
Single stage (*n* = 232)		1 [Referent]		–	–	–
Completion (*n* = 36)	0.582	0.243–1.393	0.224	–	–	–
Portomesenteric venous resection				–	–	–
No (*n* = 175)	0.998	1 [Referent]	0.994	–	–	–
Yes (*n* = 93)		0.570–1.748				
Arterial resection						
No (*n*=243)		1 [Referent]		–	–	–
Yes (*n*=25)	0.461	0.153–1.392	0.170	–	–	–
Multivisceral resection						
No (*n*=222)		1 [Referent]		–	–	–
Yes (*n*=46)	2.086	1.078–4.038	**0.029**	–	–	–
Intraoperative GVC^a^						
No (*n* = 215)		1 [Referent]			1 [Referent]	
Yes (*n* = 53)	1.957	1.041–3.677	**0.037**	2.207	1.142–4.268	**0.019**
TP/year						
<10 (*n* = 40)		1 [Referent]			1 [Referent]	
10–20 (*n* = 37)	0.517	0.202–1.325	0.169	0.580	0.224–1.502	0.262
> 20 (*n* = 191)	0.388	0.191–0.785	**0.009**	0.360	0.175–0.738	**0.005**

Bold value indicates statistical significance (*p* < 0.050)

OR, odds ratio; CI, confidence interval; BMI, body mass index (kg/m^2^); ASA-PS, American Society of Anesthesiologists–Physical Status; TP, total pancreatectomy; GVC, gastric venous congestion

^a^GVC during index surgery, so GVC during the postoperative course is not included

### Gastric Venous Congestion

Logistic regression analysis identified PVR (OR, 2.103; 95% CI, 1.034–4.278) and left coronary vein ligation (OR, 11.858; 95% CI, 5.772–24.362) as independent predictors for the development of GVC. Because all the patients who underwent a left coronary vein ligation also underwent a concomitant splenic vein ligation, the ligation of both veins was not tested as a separate independent variable (Table 5 presents the logistic regression analysis of predictors for GVC).

**Table 5:** Predictors for gastric venous congestion

	Univariable analysis			Multivariable analysis		
Variable	OR	95% CI	*p* Value	OR	95% CI	*p* value
Hypertension						
No (*n* = 157)		1 [Referent]		–	–	–
Yes (*n* = 111)	1.361	0.755–2.451	0.305	–	–	–
Peripheral vascular disease						
No (*n* = 249)		1 [Referent]		–	–	–
Yes (*n* = 19)	0.677	0.190–2.410	0.547	–	–	–
Ischemic heart disease						
No (*n* = 247)		1 [Referent]		–	–	–
Yes (*n* = 21)	2.487	0.977–6.332	0.056	–	–	–
Smoking						
No (*n* = 223)		1 [Referent]		–	–	–
Yes (*n* = 45)	0.768	0.336–1.756	0.531	–	–	–
TP type						
Single-stage (*n* = 232)		1 [Referent]		–	–	–
Completion (*n* = 36)	0.090	0.012–0.670	**0.019**	–	–	–
Portomesenteric venous resection						
No (*n* = 175)		1 [Referent]			1 [Referent]	
Yes (*n* = 93)	3.808	2.072–6.999	**< 0.001**	2.103	1.034–4.278	**0.040**
Arterial resection						
No (*n* = 243)		1 [Referent]		–	–	–
Yes (*n* = 25)	1.189	0.451–3.131	0.726	–	–	–
Spleen preservation^a^						
No (*n* = 233)		1 [Referent]		–	–	–
Yes (*n* = 35)	0.093	0.012–0.695	**0.021**	–	–	–
Left coronary vein ligation^b^						
No (*n* = 210)		1 [Referent]			1 [Referent]	
Yes (*n* = 58)	14.727	7.342–29.542	**< 0.001**	11.858	5.772–24.362	**< 0.001**

Bold value indicates statistical significance (*p* < 0.050)

OR, odds ratio; CI, confidence interval; TP, total pancreatectomy

^a^Splenic vein is preserved in all patients with spleen preservation, ^b^In all patients who underwent a left coronary vein ligation, the splenic vein also was ligated.

A subanalysis was performed within the cohort of patients who experienced intraoperative GVC, comparing the patients who underwent a (sub)total gastrectomy for GVC and those who had conservative treatment. A left coronary vein resection (with or without splenic vein ligation) tended to be performed more often for the patients who needed a (sub)total gastrectomy (*n* = 22/29 [75.9%] vs* n* = 12/24 [50.0%]; *p* = 0.051). Also, extended vascular resection tended to be performed more frequently in this group (*n* = 22/29 [75.9%] vs* n* = 12/24 [50.0%]; *p* = 0.051), particularly arterial resection (*n* = 6/29 [20.7%] vs* n* = 0/24 [0%]; *p* = 0.027) and concomitant PVR with arterial resection (*n* = 5/29 [17.2%] vs* n* = 0/24 [0%]; *p* = 0.056) (Appendix 2 presents more procedural details for both groups).

Although the number of patients who underwent a vascular resection in the (sub)total gastrectomy group was higher, the rates for morbidity (*n* = 10/29 [34.5%] vs *n* = 11/24 [45.8%]; *p* = 0.400) and 90-day mortality (*n* = 2/29 [6.9%] vs* n* = 1/24 [4.2%]; *p* = 1.000) were similar between the patients with and without (sub)total gastrectomy (Appendix 3 presents the postoperative outcomes in both groups).

## Discussion

This retrospective high-volume single-center study demonstrated a 21% incidence of GVC among 268 patients who underwent an elective single-stage/completion TP. GVC was identified particularly during index surgery (93%), and GVC was managed intraoperatively with a (sub)total gastrectomy in 55% of patients. Intraoperative GVC was predictive for major morbidity, although no significant difference was seen in 90-day mortality between the patients with and without GVC (5% vs 3%). The predictors for GVC were PVR and left coronary vein ligation.

This is only the second study that investigated potential risk factors, outcome, and management of GVC among consecutive patients who underwent TP. The incidence of GVC in the current study was similar to that in a retrospective series of 585 patients from the Heidelberg study (21% vs 28%), wherein GVC was observed mostly during index surgery (94%), as in the current study (93%).^21^ In contrast, Barbier et al.^22^ reported a 9% incidence of intraoperative GVC in a single-center cohort of 56 patients. possibly due to a lower rate of PVR. Similar findings were observed in another retrospective single-center study of 38 patients who underwent TP, among whom the incidence was 5%, although 63% of the patients underwent a PVR.^20^ Furthermore, the wide range of GVC incidence also could have been caused by different definitions. Whereas left coronary vein ligation and PVR were identified as predictors for GVC in the current study, Loos et al.^21^ demonstrated that preoperative chemo(radio)therapy, single-stage elective TP, and splenectomy increased the risk for GVC but, in contrast, not PVR.

A remarkable difference existed in GVC management between the current cohort and the Heidelberg group. In the Heidelberg group, all the patients with intraoperative GVC underwent a partial gastrectomy, whereas only 55% of the patients in the current study needed a partial or total gastrectomy (defined as either subtotal or total gastrectomy). Of the 154 patients with intraperative GVC in the Heidelberg cohort, 20 (13%) underwent a relaparotomy because of postoperative GVC. In their overall cohort, the incidence of postoperative GVC was 5%, all managed with partial or total gastrectomy. In contrast, only 1.5% of the patients in the current cohort had GVC diagnosed postoperatively, with no patient requiring a relaparotomy. These differences in intraoperative GVC management could be explained by the lack of standardization in GVC diagnosis, hypothetical differences in the severity of GVC (data not available) and/or variety in the indication, and lower threshold and local traditions for performance of a gastrectomy. Based on the higher rate of gastrectomies for intraoperative GVC in the Heidelberg study, it could be argued that this lower threshold for a partial gastrectomy reduces the risk for postoperative GVC. However, the opposite seems to be true. Furthermore, the higher rate of postoperative GVC reported by Loos et al.^21^ may have resulted from an ischemic component of the stomach after celiac axis resections, performed for 4% of the patients.

In our institution, the decision to perform a (sub)total gastrectomy for intraoperative GVC was based mainly on the severity and extent of GVC by visual judgement. For 93% of the patients who underwent gastrectomy, a subtotal gastrectomy was considered sufficient because the proximal stomach received arterial blood flow from the aortic esophageal arteries. However, it also is plausible that the extent of gastric resection depended on the surgeon’s individual preference and experience considering that a gastroenteroanastomosis is not technically as demanding as an esophagoenteroanastomis. This further illustrates the need for a standardized severity-based classification to guide the management of GVC.

Whereas the 90-day mortality among the patients with intraoperative GVC was similar between the current study and the Loos et al.^21^ study (6% vs 6%), the mortality rate was much lower among the few patients with postoperative GVC in the current Karolinska cohort (0% vs 24%). Loos et al.^21^ demonstrated that a relaparotomy with gastrectomy was an independent predictor for 90-day mortality. However, their logistic regression analysis was limited by testing multiple independent parameters for just a few mortality events. Therefore, the impact of relaparotomy for GVC on mortality remains unclear.

Although the current study provided valuable new insights into the risk factors, management, and outcome of GVC after TP, more evidence is required, particularly about intraoperative detection, management, and prevention of postoperative GVC. Various techniques to manage GVC intraoperatively have been described concerning either partial/total gastrectomy or reconstruction of the gastric venous outflow. A patient reported by Sandroussi and McGilvray^16^ underwent ligation of the inferior mesenteric vein (that drained into the superior mesenteric vein) so an end-to-end reconstruction could be created with the left coronary vein that managed the GVC successfully. The Heidelberg group published various reconstruction techniques including (1) end-to-side reconstruction of the left coronary vein on the portal vein,^33^ (2) reconstruction of the splenic vein on either the portal vein, inferior caval vein, or left renal vein (splenorenal shunt) in case of spleen preservation,^34,35^ (3) reconstruction of the left coronary vein on the left renal vein, and (4) reinsertion of the right gastroepiploic/coronary vein.^35^ An observational bi-center study including 92 patients who underwent TP showed that spleen-preservation with its vasculature also could be considered, even in case of PVR, preserving the left gastroepiploic and short gastric veins.^36^ In a case report, Hishida et al.^17^ described preservation of the gastrocolic trunc and right gastroepiploic vein to avoid GVC. On the other hand, others have performed partial/(sub)total gastrectomy to manage GVC instead,^20,21^ as was done in the current study, although 44% of the patients were treated without any gastric resection or gastric venous reconstruction.

The prospective observational single-center GENDER study might provide further insight into the prevention and management of GVC after TP with concomitant left coronary vein ligation.^37^ In this study, gastric venous drainage will be systematically assessed pre-,^38^ intra-, and postoperatively, then adressed by venous outflow reconstuction, followed by a partial/total gastrectomy when management of GVC by gastric venous outflow reconstruction fails. This systematic approach could reduce the need for gastric resection as well as postoperative relaparotomies for delayed GVC. Possibly, this could improve the associated morbidity and mortality. Furthermore, it could lower the need for gastric resections and associated burdens such as increased morbidity/mortality, potential longterm malnutrition, and reduced quality of life.^39,40^ However, the patency and efficacy of venous reconstruction need to be studied with larger cohorts in relation to the risk for delayed postoperative GVC and left-sided portal hypertension. Currently, it is difficult to foresee whether the potential benefit of venous outflow reconstruction outweighs the potential risks as well as the short- and long-term side effects of a (sub)total gastrectomy.

Although TP is not commonly performed with a large variety exists in its use based on sparse evidence,^3^ GVC after TP seems to be an underestimated complication with a high incidence and an association with morbidity. In the current era, 23% of patients with locally advanced pancreatic cancer can undergo a resection after multi-agent induction chemotherapy.^41,42^ Hereby, extended resections are often required, including extensive PVR^43–45^ and sometimes TP for technical and/or oncologic reasons.^5,46,47^ As a consequence, surgeons currently might face GVC more frequently. Therefore, a need exists for standardization of terminology, including a severity-based classification for GVC to support clinical decision-making, prognostication, and research on the management of GVC.

The results from the current study have to be interpreted in the light of some limitations. First, the retrospective methodology of this study might have resulted particularly in an underestimation of the GVC incidence, especially due to underreporting of less severe GVC. Second, it was not possible to reliably collect the precise portomesenteric venous anatomy because of the study’s retrospective nature. The venous diameter of the portomesenteric, splenic, and gastric branches as well as anatomic deviations of the inferior mesenteric vein might be of relevance for the risk of GVC and left-sided portal hypertension. The impact of these parameters needs to be studied in future prospective series. Third, although the operation reports were detailed, underreporting about vascular anatomy and ligation/resection might have influenced the results. Fourth, it was not possible to properly investigate the risk of treating GVC with a combined splenic and left coronary vein ligation versus left coronary vein ligation with preservation of the splenic vein because all patients with left coronary vein ligation have always undergone concomitant resection of the splenic vein. Fifth, the impact of GVC on mortality could not be investigated due to the low number of events. Future prospective studies must investigate long-term sequelae of impaired gastric venous outflow, such as variceal bleedings and reduced gastrointestinal function. The major strength of the current study was its reduction of the knowledge gap. This is the second study conducted to investigate predictors, management, and outcome of GVC after TP while also analyzing the impact of various cardiovascular comorbidities on the risk for GVC.

## Conclusion

In conclusion, GVC after TP is rather common, occurring in approximately one of five patients, with GVC during index surgery predicting postoperative major morbidity but not translating into higher mortality. This complication should be considered in the preoperative decision-making and patient counseling. Left coronary vein ligation and PVR are predictive for GVC, requiring vigilance during and after surgery, for instance by additional intra- and postoperative diagnostics (e.g., gastroscopy, indocyanine green fluorescence), and a lower threshold for surgical interventions (e.g., [sub]total gastrectomy, reconstruction of gastric venous outflow). Importantly, it seems that a gastric resection is not always needed. More evidence on prevention, examination, classification, and management of GVC is needed.

## Appendix

### Appendix 1

**Table:** Procedural details: single-stage elective TP

Variable	All patients (*n* = 232)	Without GVC (*n* = 176)	With GVC (*n* = 56)
Intraoperatively decided, *n* (%)	148 (63.8)	108 (61.4)	40 (71.4)
Radicality reasons	87 (58.8)	59 (54.6)	28 (70.0)
Technical reasons	56 (37.8)	44 (40.7)	12 (30.0)
Vascular resection	17 (30.4)	10 (22.7)	7 (58.3)
High-risk pancreatico-enterostomosis	36 (64.3)	32 (72.7)	4 (33.3)
Others	3 (5.4)	2 (4.5)	1 (8.3)
Others	5 (3.4)	5 (2.8)	0 (0)

TP, total pancreatectomy; GVC, gastric venous congestion

### Appendix 2

**Table:** Intraoperative gastric venous congestion: procedural details

	(Sub)total gastrectomy		
Variable	Yes	No	*p* value
	(*n* = 29)	(*n* = 24)	
TP type, *n* (%)			1.000^a,b^
Single-stage elective	28 (96.6)	24 (100)	
Preoperatively planned	6 (20.7)	9 (37.5)	
Intraoperatively decided	22 (75.9)	15 (62.5)	
Elective completion	1 (3.4)	0 (0)	
Pylorus preservation, *n* (%)	–	2 (8.3)	–
Spleen preservation, *n* (%)	0 (0)	1 (4.2)	0.453^a^
Ligation splenic vein, *n* (%)	29 (100)	23 (95.8)	0.453^a^
Ligation left coronary vein, *n* (%)	22 (75.9)	12 (50.0)	0.051^c^
Ligation splenic vein + left coronary vein, *n* (%)	22 (75.9)	12 (50.0)	0.051^c^
Vascular resection, *n* (%)	22 (75.9)	12 (50.0)	0.051^c^
Portomesenteric venous resection			0.062^a^
Type 1−2	1 (3.4)	3 (12.5)	
Type 3−4	20 (69.0)	9 (37.5)	
Arterial resection^d^	6 (20.7)	0 (0)	**0.027** ^a^
Hepatic artery	5 (17.2)	0 (0)	0.056^a^
Superior mesenteric artery	1 (3.4)	0 (0)	1.000^a^
Venous + arterial resection	5 (17.2)	0 (0)	0.056^a^
Multivisceral resection other than gastric resection, *n* (%)	2 (6.9)	2 (8.3)	1.000^a^

Bold value indicates statistical significance (*p* < 0.050)

TP, total pancreatectomy

^a^Fisher’s exact test, ^b^Single-stage elective vs elective completion, ^c^Chi-square test, ^d^All arterial resections are reconstructed

### Appendix 3

**Table:** Intraoperative gastric venous congestion: postoperative outcome

	(Sub)total gastrectomy		
Variable	Yes	No	*p* value
	(*n* = 29)	(*n* = 24)	
DGE grade B/C, *n* (%)^a^	4 (14.8)	2 (8.3)	0.671^b^
PPH grade B/C, *n* (%)	2 (6.9)	1 (4.2)	1.000^b^
Bile leakage grade B/C, *n* (%)	0 (0)	0 (0)	–
Major morbidity, *n* (%)	10 (34.5)	11 (45.8)	0.400^c^
Relaparotomy	3 (10.3)^d^	2 (8.3)^e^	1.000^b^
Organ failure			0.224^b^
Single-organ failure	2 (6.9)	5 (20.8)	
Multi-organ failure	0 (0)	0 (0)	
Multiple major morbidity (> 1), *n* (%)	3 (10.3)	4 (16.7)	0.688^b^
Postoperative hospital stay (days) (median, IQR)	12 (10–15)	10 (8–18)	0.548^f^
Intensive care stay,* n* (%)	3 (10.3)	2 (8.3)	1.000^b^
In-hospital mortality, *n* (%)	0 (0)	0 (0)	–
90-day mortality, *n* (%)	2 (6.9)	1 (4.2)	1.000^b^

Bold value indicates statistical significance (*p* < 0.050)

DGE, delayed gastric emptying; PPH, postpancreatectomy hemorrhage; IQR, interquartile range

^a^Patients who underwent a total gastrectomy (*n* = 2) are excluded, ^b^Fisher’s exact test, ^c^Chi-square test, ^d^Indications for relaparotomy were wound dehiscence (*n* = 2) and PPH (*n* = 1), ^e^Indication for relaparotomy was wound dehiscence (*n* = 2), ^f^Mann-Whitney *U* test
